# Common analysis pitfalls in longitudinal ctDNA studies: lead time, sensitivity and immortal time bias

**DOI:** 10.1016/j.ebiom.2026.106407

**Published:** 2026-07-27

**Authors:** Dominik Hlauschek, Nadia Dandachi, Michael Gnant, Heather Parsons, Angela DeMichele, Ellen Heitzer, Gabriel Rinnerthaler, Marija Balic

**Affiliations:** a Division of Oncology, Department of Internal Medicine, Medical University of Graz, Graz, Austria; b Austrian Breast and Colorectal Cancer Study Group [ABCSG], Vienna, Austria; c Comprehensive Cancer Center, Medical University of Vienna, Vienna, Austria; d Clinical Research Division, Fred Hutchinson Cancer Center, Seattle, WA, USA; e Medical Oncology, Perelman School of Medicine, University of Pennsylvania, Philadelphia, PA, USA; f Institute of Human Genetics, Diagnostic & Research Center for Molecular BioMedicine, Medical University of Graz, Graz, Austria; g Division of Oncology, Hillmans Cancer Center, University of Pittsburgh, Pittsburgh, PA, USA

**Keywords:** ctDNA, MRD, Immortal time bias, Lead time, Sensitivity, Specificity

## Abstract

Circulating tumour DNA (ctDNA) has emerged as one of the most promising biomarkers in oncology, with potential applications from detection of minimal residual disease (MRD) and diagnosis to prognosis, treatment guidance, and response monitoring. This personal view critically examines recurring methodological and statistical pitfalls in ctDNA studies with longitudinal measurements. We highlight important issues with lead time definition and demonstrate that performance metrics like sensitivity and specificity are inherently time-dependent. Furthermore, we show why immortal time bias arises when classifying patients incorrectly as always positive, based on becoming positive during surveillance in MRD related studies. With a combination of toy data examples, simulations, and re-analysis of published data we provide practice-oriented guidance to support the design, analysis, and reporting of ctDNA trials. This viewpoint paper aims to sensitise the community and raise awareness of pitfalls in longitudinal ctDNA analysis, rather than offer definitive solutions–more evidence is needed first.

**Search strategy and selection criteria**

The authors identified the primary literature featured in this Personal View over the years while conducting research on ctDNA studies (with a focus on Breast Cancer). For identifying relevant ctDNA studies covering sensitivity and specificity specific searches on PubMed using the terms “Sensitivity” OR “Specificity” OR “ctDNA” OR “MRD” OR “time-dependent” OR “ROC” were performed. All relevant ctDNA studies regardless of retrospective or prospective in nature were included. No specific exclusion criteria were applied. In addition, Perplexity’s® “Deep Research” mode was used to search for ctDNA studies reporting time-dependent sensitivity. Abstracts and meeting reports of ctDNA studies were reviewed during literature research, but none were included in this personal view as it was not applicable, or the original article was used instead. All cited papers were evaluated for relevance and quality before inclusion.

## Introduction

Circulating tumour DNA (ctDNA) has emerged as one of the most promising biomarkers in oncology, with potential applications spanning the entire continuum of the cancer care pathway–from early detection and diagnosis to prognosis, treatment guidance, and response monitoring.^1^

Despite this broad potential, the current clinical validity of ctDNA testing remains most established in the prognostic and response-monitoring domains. In the post-surgery setting, ctDNA positivity after curative-intent therapy strongly correlates with risk of relapse. However, as ctDNA becomes increasingly integrated into both clinical trials and routine oncology practice, critical questions persist: How should ctDNA test results inform clinical decision-making? What level of assay performance–sensitivity, specificity, and lead time–is required for reliable clinical interpretation and actionability?

Addressing these questions requires robust assay performance and understanding of methodological and analytical limitations. Variability in sample handling, processing, sequencing, error-correction, and interpretation thresholds influences assay accuracy.^2^, ^3^, ^4^ Without rigorous validation and careful statistical design, ctDNA assays may risk yielding misleading results.

In this commentary, we critically examine three core aspects of ctDNA analysis that are central to establishing clinical utility in the surveillance setting: i) Lead time–the interval between ctDNA detection and clinical relapse; ii) Diagnostic accuracy metrics–including sensitivity, specificity, and predictive values; and iii) Estimation of relapse risk associated with ctDNA positivity.

We identify common methodological issues and biases in ctDNA research, using examples from toy data, simulations, and published studies like the I-SPY2 trial. We also provide literature references and example SAS and R code to implement appropriate methods. Our recommendations focus on ctDNA as a prognostic and predictive biomarker in the post-surgery surveillance setting when the tumour was removed but also apply to metastatic and early-detection scenarios.

## Lead time–from ctDNA detection to clinical relapse

Multiple studies, systematic reviews, and meta-analyses have attempted to quantify the lead time between ctDNA detection (often in a “minimal residual disease” or post-treatment monitoring setting) and overt clinical or radiographic relapse.^5^, ^6^, ^7^, ^8^, ^9^ The results are heterogeneous given “convenience sampling” with different start times in the disease trajectory, but some patterns emerge. The lead time–the interval from the initial detectable ctDNA signal to a clinically evident disease recurrence–is foundational for multiple reasons. It defines the potential window for earlier therapeutic intervention, and informs optimal surveillance scheduling, and the design of future clinical trials. However, estimating lead time reliably demands rigorous consistency in definitions and methodological care. Without these, comparisons across studies risk conflating fundamentally different constructs.

### Lead time definition

In its broadest sense, lead time represents the interval between the first biomarker-detectable sign of disease and its clinical or radiological confirmation. In the context of ctDNA monitoring, this concept translates to the interval between the first ctDNA-positive test and the clinical or radiological detection of relapse. While this seems straightforward, several important methodological and interpretative challenges arise.

**Fig. 1:**
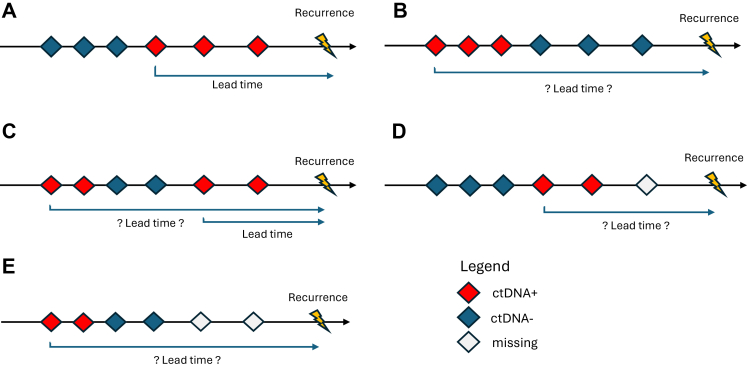
Examples of ctDNA test results and their implications for lead time estimation. A) ctDNA is detected 3 time-points ahead of relapse with all following ctDNA tests being positive. Lead time can be calculated from first detection of ctDNA. B) ctDNA is cleared after 3 consecutive positive ctDNA tests and stays non-detectable until relapse. Time from first positive ctDNA test may no longer be considered a lead time for relapses. C) ctDNA is initially detected, followed by two timepoints without ctDNA detection, and then detected again at a later timepoint before relapse. Only the time from the later timepoint testing may be considered a lead time of relapse. D) and E) show scenarios where the lead time is no longer clear as ctDNA positive testing followed missing test results.

In simple scenarios, where ctDNA remains persistently positive from first detection until relapse (Fig. 1a), defining lead time from the first positive test is appropriate. This approach aligns with the definition proposed by the Blood Profiling Atlas in Cancer (BloodPAC) Consortium, which describes lead time as “the difference in time between the first positive ctDNA result and subsequent clinical or radiologic evidence of disease recurrence or progression.”^10^ However, real-world ctDNA dynamics are often more complex. Some patients exhibit transient positivity followed by one or more negative results (Fig. 1b). Defining lead time from the initial positive result in such cases may substantially overestimate the true interval and misattribute biological relevance. The subsequent recurrence may, in fact, be unrelated to the earlier ctDNA signal -particularly if intervening tests were negative, suggesting either genuine molecular clearance or assay variability. This raises a further interpretative dilemma: if a patient becomes ctDNA-positive, then negative on several subsequent tests, and later develops overt metastasis, does the “lead time” span that entire interval? Possibly not. In this context, we suspect the earlier signal may represent either a false positive or an analytical artifact rather than a true indicator of persistent residual disease and prospective ongoing trials will hopefully address this phenomenon. This may be especially true for low ctDNA levels and may become more relevant in the future with more sensitive assays which can detect very low levels. A more informative pattern is one of re-emergent or sustained ctDNA positivity after initial clearance (Fig. 1c).

The complexity deepens when considering the interplay between biological and analytical variability. Transient ctDNA negativity may reflect true biological suppression (e.g., therapy-induced clearance) or methodological limitation (e.g., subthreshold detection, sampling variation, or assay failure). If the latter is true–i.e., the assay simply failed to detect ctDNA that was biologically present–then measuring lead time from the first positive test remains conceptually valid. However, if molecular clearance is genuine and the later recurrence arises from new or dormant clones, the earlier signal is likely biologically unrelated and should not contribute to lead-time estimation.

Lead time estimation also highly depends on the sampling frequency as well as timing of disease monitoring. Regardless of the lead time definition, the sampling density does matter, and misclassifications may be reduced with more frequent sampling.

Considering the issues above, one possible definition may be the interval between the *first* positive ctDNA result *NOT followed by a ctDNA negative result* and subsequent clinical or radiological evidence of disease recurrence or progression. However, this definition also has its limitations, as it is affected by potential hindsight bias and misclassifications. This is particularly true when ctDNA analyses are performed at longer intervals (e.g., every 6–12 months), since with longer intervals it cannot be ruled out that intermittent results might be negative. Furthermore, this applies especially to situations where no ongoing treatment is being administered, since—for example—in late-intervention studies, temporary clearance cannot be ruled out.

It seems fair to say that this topic remains unresolved, and based on today’s knowledge, no explicit lead time definition can be recommended. Further research is necessary, rather than merely reporting lead times without providing detailed information and explicit considerations.

**We recommend** investigating different lead time definitions instead of just time from first ctDNA positive test until relapse. We also recommend reporting the sampling density whenever feasible, performing sensitivity analysis considering that ctDNA assays may report spurious test results (e.g., no detection of ctDNA due to methodological limitations) and investigating different definitions of lead time.

### Lead time estimation

Currently, methods used to estimate lead time are often incompletely described. Frequently, studies report the simple median lead time among patients who experienced clinical relapse after ctDNA detection. However, this approach can substantially underestimate the true median because it excludes censored observations–patients who were ctDNA-positive but had not relapsed by the end of follow-up.

Lead-time estimation is fundamentally a time-to-event problem–that is, the time from ctDNA detection (event A) to clinical or radiological recurrence (event B). Consequently, its analysis should follow the same statistical principles as standard survival analysis. Some ctDNA-positive patients may not yet have experienced recurrence because of limited follow-up, or loss to follow-up. Excluding such cases from analysis can lead to systematic underestimation of the true lead time.

Consider a study including seven ctDNA-positive patients, of whom five relapsed with observed lead times of 3.1, 5.3, 6.4, 7.9, and 9.3 months, while two had not relapsed by study end and were censored at 10 months. When censoring is properly accounted for, the estimated median lead time is approximately 7.9 months (i.e., middle value of those 7 patients). In contrast, calculating the median using only the five relapse events underestimates the true value (6.4 months), demonstrating how exclusion of censored observations can bias lead-time estimation.

This bias becomes more pronounced as the proportion of censored patients increases. A small simulation study (10,000 simulations; true median = 12 months) demonstrated progressively greater underestimation of the median lead time with higher censoring rates. The true lead time was underestimated by more than 4 months when the censoring rate was 30% within 1 year (Supplementary Table S1). Therefore, accurate estimation of lead time requires statistical methods that appropriately handle censoring such as Kaplan–Meier estimation (time from positive test until event/last follow up), rather than relying solely on observed events.

Missing ctDNA measurements add complexity. Missed blood draws can affect lead-time estimation (Fig. 1d and e) and should be addressed analytically. Multiple imputation or censoring at missing tests may be used if justified. In either case, informative censoring should be considered, and sensitivity analyses are recommended.

**We recommend** that lead time in ctDNA studies be estimated using survival analysis methods rather than simple medians of observed events. Censoring and competing risks must be appropriately handled, and missing ctDNA tests addressed.

### Implementation examples

For an example of the median lead time estimated with Kaplan Meier approach see the results of the c-TRAK TN trial.^11^ Unfortunately, the authors do not provide details about their implementation of the Kaplan Meier estimation. Furthermore, see the Supplementary materials section “Lead time calculation example” for a detailed walkthrough using a published ctDNA data set.

## Diagnostic accuracy: time-dependent performance of ctDNA assays

To assess and compare ctDNA assays accuracy, several measures are commonly reported in the liquid biopsy literature. Most common measures include sensitivity, specificity, positive predictive value (PPV), and negative predictive value (NPV).

It is important to distinguish between *analytical* and *clinical* measures. *Analytical* sensitivity is often used to describe the ability of an assay to detect ctDNA in the blood, whereas clinical sensitivity refers to the ability of the assay to detect disease recurrence. We focus on the latter.

### Definition of performance metrics

The four primary diagnostic performance metrics–sensitivity, specificity, PPV, and NPV along with their conceptual definitions and interpretations, are summarised in Supplementary Table S2. Formal statistical expressions for these measures are also provided in the Supplementary Material. These definitions already imply that diagnostic performance is time dependent. Unlike a static diagnostic test–where disease status is known at the time of testing–recurrence in the adjuvant setting occurs after ctDNA measurement. Consequently, all four metrics evolve during follow-up as relapses occur.

**Fig. 2:**
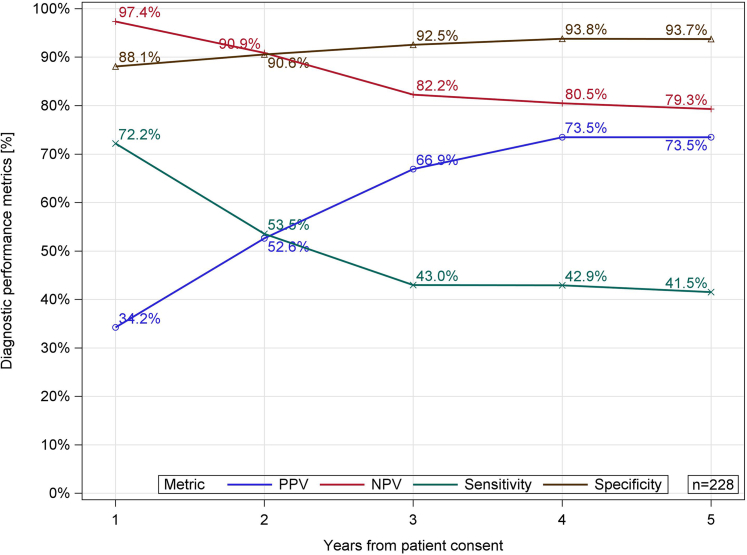
Positive predictive value (PPV), Negative predictive value (NPV), Sensitivity and Specificity of pre-surgical ctDNA status for the first 5 years from patient consent (=prior to any neoadjuvant treatment) using the I-SPY 2 data. Estimated using Kaplan Meier methodology. See page 1 of Supplementary File for estimation details and SAS code to replicate this figure.

To illustrate the time-dependent behaviour of diagnostic performance we re-analysed a published ctDNA dataset from the I-SPY2 Breast Cancer trial^12^ using just the pre-surgery time point (T3; n = 228). In short, I-SPY2 is a neoadjuvant phase 2 adaptive platform trial testing several investigational drugs (alongside standard chemotherapy) in high-risk Breast Cancer patients. Fig. 2 shows the trend over time of the 4 metrics. The PPV increases steadily with a longer follow up, as more and more events occur. Conversely, the NPV is highest early in follow-up—when few recurrences have yet occurred and gradually declines over time. Clinical sensitivity and specificity do not exhibit a consistent temporal trend. However, sensitivity may frequently decrease, while specificity tends to increase over time, reflecting that ctDNA positivity is typically more strongly associated with imminent (shorter-term) than delayed recurrence and patients who are ctDNA-negative at early timepoints tend to relapse rather later in time.

Together, these examples emphasise the time-varying nature of diagnostic accuracy in ctDNA studies. Reporting sensitivity, specificity, PPV, or NPV at a single, fixed time point may obscure these temporal dynamics and hinder meaningful comparison across assays or clinical studies.

### Estimation

As stated above, accurate estimation of ctDNA assay performance metrics requires explicit consideration of their time-dependent nature. Clinically meaningful evaluation points should be defined *a priori,* and censoring due to unequal follow-up or loss to follow-up must be properly handled and reported. A common but flawed approach treats relapse as a static binary outcome and derives these metrics using simple proportions (e.g., sensitivity = ctDNA-positive/total relapsed).^5^^,^^13^, ^14^, ^15^, ^16^ Such estimates are not generalisable because they depend on the specific follow-up duration within a study.

To illustrate this, we re-analysed the same I-SPY2 ctDNA dataset^12^ at the pre-surgery time point adding further loss to follow up censoring (Table 1). Naive, time-insensitive estimates (Table 1, column 2) were compared with results from three simulation scenarios introducing varying censoring rates, each repeated 5000 times (see Supplementary Methods). All four metrics changed with censoring: PPV decreased and NPV increased as censoring rose, while sensitivity and specificity were moderately affected when censoring differed between ctDNA-positive and ctDNA-negative groups. These findings underscore the need for time-to-event methods, such as Kaplan–Meier estimation, to obtain unbiased estimates.

**Table 1:** Naively estimated performance metrics of pre-surgical ctDNA data from I-SPY2.^12^

Statistic	Naive estimate (no additional censoring)	Artificial yearly censoring rate for ctDNA positive and non-detected patients		
	No additional censoring	5%/5% yearly censoring^a^	10%/10% yearly censoring^a^	20%/5% yearly censoring^a^
Sensitivity	44.3%	45.1%	45.9%	39.3%
Specificity	93.4%	92.5%	91.7%	89.8%
PPV	71.1%	66.0%	61.2%	52.4%
NPV	82.1%	83.9%	85.5%	83.9%

Simple proportions—not considering time-dependency—were used to calculate the metrics (i.e., naive estimate column). Three different simulation scenarios were used to introduce artificial censoring with varying yearly rates for ctDNA positive and ctDNA non-detected patients. In the first simulation scenario the yearly censoring rate was 5% for ctDNA positive and non-detected patients. In the second scenario the rate was increased to 10% for both, and in scenario 3 imbalanced censoring rates of 20% and 5% were used for ctDNA positive vs. non-detected patients, respectively. 5000 Simulations were run and numbers presented are the averages. Details of the simulation can be found in the supplement.

a Yearly censoring rates for ctDNA-positive/ctDNA-non-detected patients.

This problem has long been recognised in the statistical literature. Heagerty et al. (2000) first generalised static sensitivity and specificity into time-dependent forms.^17^ Subsequent work has refined these models, as reviewed by Kamarudin et al.,^18^ and shown by Antolini et al.^19^ to be applicable to binary biomarkers such as ctDNA. While most studies address single testing time points, others extend these frameworks to serial testing–an approach well-suited for ctDNA surveillance.^20^

Interested readers, who want to get started with time-dependent sensitivity and specificity estimation R packages like survivalROC or timeROC may be good starting places. In SAS, these metrics may be estimated with the option ROCOPTIONS in the PHREG procedure. Furthermore, Bansal and Heagerty provide a detailed tutorial for application of those methods including the mentioned software packages.^21^ However, special considerations are warranted as most software options for time-dependent sensitivity estimation are intended for continuous biomarkers. Slight code adaptions may be necessary. However, the formulas provided in the appendix can also be coded manually. An example of SAS code (which was also used for the I-SPY2 re-analysis) using those formulas can be found in the Supplementary material.

Estimation of PPV and NPV is more straightforward but still requires survival analysis. Both can be derived using Kaplan–Meier estimation for ctDNA-positive and negative cohorts, with time measured from the test of interest (for detailed examples see Fig. 2 and Supplementary Table S3).

Beyond enabling unbiased estimation, these time-dependent approaches can inform clinical implementation, such as identifying optimal ctDNA thresholds or test intervals and length of follow up needed to assess the performance of assays adequately. For example, in the I-SPY2 study, pre-surgical ctDNA sensitivity declined from 72% at year 1–43% at year 3, indicating that predictive power diminishes over time and repeat testing becomes necessary, whereas specificity remained high (Fig. 2).

**We recommend** estimating diagnostic metrics in ctDNA studies as time-dependent measures using survival analysis, not static proportions. Censoring, competing risks, and clinically relevant timepoints must be accounted for and transparently reported to ensure unbiased and comparable results (see Table 2).

**Table 2:** Recommendations for future ctDNA studies at a glance.

Quantity	Recommendations
Lead time	- Clearly specify the quantity subject to estimation. - Estimation should consider censoring. - Median (and other quantiles) can be estimated with Kaplan Meier approach.
Sensitivity, Specificity, PPV, and NPV	- Time dependence should be considered. - Clinically relevant time points should be selected for those metrics. - Loss to follow up prior to the time points should be considered. - Sensitivity and specificity can be estimated with time-dependent ROC software. - PPV and NPV can be estimated with Kaplan Meier approach.
Association of ctDNA with relapse	- Consider immortal/guarantee time bias. → do not use “future” ctDNA information to create groups assuming they are fixed beginning from baseline. → Time periods during ctDNA non-detection vs. detection need to be assigned correctly to the respective groups (e.g., censor ctDNA non-detected patients who become ctDNA positive). - Use appropriate methods like for example landmark, clock-reset, Simon-Makuch, time-dependent Cox models, and Joint Survival models. - Consider competing events like death without prior recurrence for absolute risk estimation (e.g., Aalen-Johansen estimator).

### Implementation examples

Unfortunately, we were unable to find a ctDNA paper that already uses the appropriate time-dependent estimation of diagnostic metrics. Therefore, we provided an example using the I-SPY2 data, along with corresponding SAS code and formulas, to implement this approach. In order to implement the estimation in R we provided further information above.

## ctDNA and clinical outcomes: avoiding bias in risk estimation

A key question is whether ctDNA detection predicts future relapse, and how relapse risk evolves in patients with undetectable ctDNA. Both aspects are clinically relevant: ctDNA positivity may indicate the need for treatment escalation, while persistently undetectable ctDNA could support de-escalation. To optimise patient management and guide trial design, clearly defining both the clinical objectives and the corresponding risk estimands (=the population quantities which are to be estimated), is essential, as recommended by the IHC guidelines.^22^

In the post-surgery surveillance setting, many clinically relevant questions arise, but two key examples are:

1) Should ctDNA-positive patients receive more intensive or prolonged adjuvant therapy?
2) Can ctDNA-negative patients safely de-escalate certain (standard) treatments?

These two questions are interrelated and can be linked to following estimands:

1) Relapse risk *following* ctDNA detection. Either at a defined single timepoint like post-surgery/treatment or at any timepoint during surveillance.
2) Relapse risk in patients with (persistently) undetectable ctDNA.

At first glance, it may seem unnecessary to define these, however, it is crucial to do so. For example, in question 1) we are not interested in the x-year relapse risk in patients who *become* ctDNA positive *at some point during follow up,* but rather in the increase in risk after we learnt about the ctDNA positive test. The difference is clinically relevant and meaningful, and failure to distinguish them may lead to misinterpretation of risk estimates, and eventually suboptimal treatment decisions.

### Estimation

Standard methods such as Kaplan–Meier and Cox proportional hazards models are commonly used to estimate relapse risk in ctDNA studies but are prone to immortal time bias if time-dependent biomarkers are analysed as a fixed baseline variable.^23^, ^24^, ^25^ This bias occurs when periods during which relapses cannot occur are incorrectly included in the risk set. Aligning the time origin of analysis with the ctDNA assessment time point mitigates this issue, ensuring that ctDNA status temporally precedes outcome evaluation. Proper specification of the estimands determines whether immortal time bias is present and ensures interpretability of results. This is particularly important when comparing relapse risk after ctDNA detection vs. non-detection at a defined testing time point. For more details about immortal time bias see also Supplementary file.

#### Relapse risk in persistently ctDNA non-detected patients

Many ctDNA studies estimate relapse risk using Kaplan–Meier curves restricted to patients who remain ctDNA-negative at all assessments (e.g.,^26^, ^27^, ^28^, ^29^). While intuitive, this approach also introduces immortal time bias by excluding relapse-free intervals before ctDNA conversion. For instance, a patient who becomes ctDNA-positive one-year post-surgery contributes a relapse-free period that must be attributed to the ctDNA-negative group; omitting this time overestimates relapse risk among ctDNA-negative patients.

**Fig. 3:**
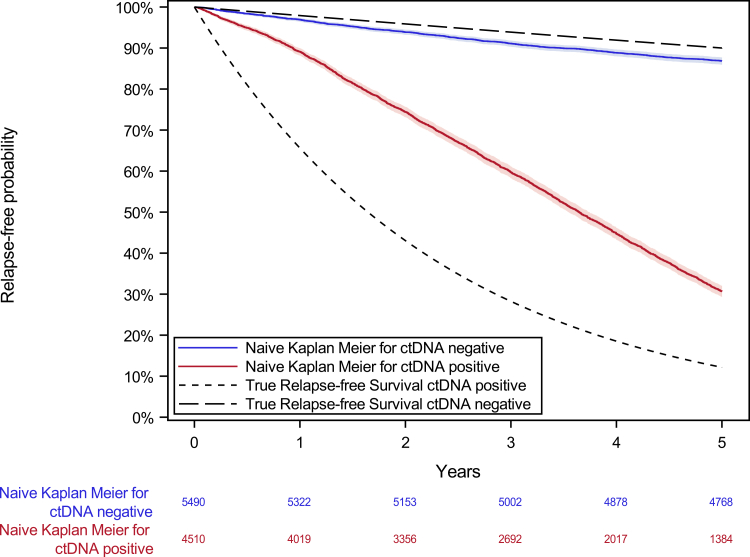
Illustration of immortal time bias. Immortal time bias in relapse-free probability estimates using naïve Kaplan Meier methodology not considering the time-dependent nature of ctDNA surveillance. A simulation with 10,000 patients and constant relapse event rates were run with the following parameters: relapse-free rates were 90% within the first 5 years for ctDNA negative patients and a hazard ratio of 20 was used for risk rate increase in positive patients. The solid lines refer to the biased estimated relapse-free rates for ctDNA non-detected patients (blue lines) and ctDNA positive patients (red lines). The dashed lines refer to the true relapse-free rates based on the simulation parameters. Simulation details and code to replicate this figure can be found in the supplement.

To illustrate this, we use a simulation example where we know the underlying truth (Fig. 3): 10,000 patients were simulated with a constant event rate over time with a 5-year relapse rate of 10% (dashed line). Patients with a positive ctDNA test had a 20-fold increased event rate (hazard ratio = 20). Further simulation details are provided in the Supplemental file including the SAS code to generate the simulation and figure. Fig. 3 solid blue line shows the estimated (naive) Kaplan Meier curve of ctDNA non-detected patients by excluding all patients who became ctDNA positive at some time prior to relapse. The risk of relapse of ctDNA non-detected patients is clearly overestimated by Kaplan Meier estimation as visualised by the true underlying survival rate (long dashed line).

A possible solution is to calculate Kaplan Meier estimates, including *all* patients and censoring those with positive ctDNA at the time of detection. This way the time periods are correctly assigned to the ctDNA non-detection group. For an example using this approach see the Supplementary material including the SAS code and Supplementary Fig. S1 which shows the unbiased estimates. However, informative censoring may need to be adjusted for in more complex real-world scenarios.

#### Relapse risk following ctDNA detection during surveillance

Naïve Kaplan–Meier analyses that include all patients who eventually become ctDNA-positive during follow-up (e.g.,^26^, ^27^, ^28^, ^29^) can yield misleading results. By attributing periods of ctDNA non-detection to the ctDNA-positive group, these analyses overestimate survival and distort the estimated relapse risk after ctDNA detection. This artifact arises because patients must remain recurrence-free long enough to be classified as ctDNA-positive, creating immortal time.

This issue can be illustrated using the same simulation example from before. For analysis, a patient was considered ctDNA positive if the ctDNA detection occurred before recurrence. Fig. 3 shows the Kaplan Meier curve of patients who became ctDNA positive prior to relapse (solid red line) vs. the true risk of relapse in dashed lines. Surprisingly, ctDNA positive patients classified by naïve group assignment have a seemingly lower relapse risk. This is solely because patients had to be recurrence-free *long enough* to become ctDNA positive. Hence, it appears as if ctDNA positive patients are doing better although we know the true recurrence risk. The same bias can occur in any analysis using the same naïve “group assignment” technique.

To circumvent this problem, several statistical methods have been proposed including landmark approaches,^30^ clock-reset approaches,^31^ extensions of the Kaplan Meier estimator,^32^^,^^33^ time-dependent Cox models,^34^^,^^35^ and joint survival models^36^ (see also Supplementary Table S4 for an overview). All these techniques have their own advantages and limitations and choosing the right method depends on the data set at hand and the scientific objectives to be addressed. For example, landmark analyses are well suited for estimating the conditional (having survived until the chosen timepoint) risk of a population based on their ctDNA status/dynamics before the chosen landmark and they are easy to implement in practice. After choosing one or more clinically relevant landmark timepoint(s) and forming the groups of interest, a simple Kaplan Meier analysis can be performed from that timepoint onward. However, this approach cannot be used to estimate the risk in patients who are persistently negative or positive, as only information from before the landmark timepoint can be used to create the groups in order to avoid immortal time bias. Nevertheless, landmark analyses are a great starting point and are clinically informative, as they provide the conditional “future” risk of recurrence based on information already available to a clinician/patient. However, if there is interest in estimating risk of patients never vs. ever detected more sophisticated methods (e.g., extensions of the Kaplan Meier estimator or clock-reset approach) should be used. The most important caveats in terms of implementation are that time-periods are correctly assigned to positive and negative periods (see also below). On the other hand, joint models are also a powerful tool as they allow simultaneous evaluation of longitudinal ctDNA trajectories and time-to-relapse outcomes, providing a dynamic view of how ctDNA patterns relate to disease progression. For instance, one could explore whether it is solely the current status of ctDNA or perhaps the trend in ctDNA levels over time that influences the risk of relapse. However, an exhaustive comparison of all the mentioned methods is out of scope of this paper. The interested reader may be directed to the following tutorials and papers^31^^,^^35^^,^^37^, ^38^, ^39^, ^40^ and to the R-packages JM,^41^ joineR,^42^ and JMBayes.^43^ Furthermore, SAS code is provided in the Supplementary material to calculate the extended Kaplan Meier estimates using PROC PHREG. See also Supplementary Fig. S1 for the unbiased risk estimates using this method. Furthermore, we strongly recommend consulting a biostatistician with expertise in these methods, as their appropriate application depends on the specific characteristics and structure of the dataset. For instance, the clock-reset approach to estimating risk in patients who become ctDNA-positive is likely appropriate only when ctDNA positivity tends to persist after first detection. If the ctDNA status often changes from positive to negative (e.g., due to effective therapy) the risk estimated with the clock-reset approach may not be very clinically informative. Unfortunately, an exhaustive list of all potential considerations cannot be provided, as these will always depend on the specific characteristics of the dataset. Finally, all these methods have their own assumptions (e.g., proportional hazards for Cox modelling or semi-Markov for clock-reset) that should be checked and reported.

A very simplified illustration and explanation of why immortal time bias occurs in ctDNA studies can also be found in the Supplementary file (Supplementary Fig. S2).

**We recommend** defining the estimand explicitly and applying analytical strategies that avoid immortal time bias. Time-at-risk must be accurately assigned to ctDNA-negative and ctDNA-positive periods (see Table 2).

### Implementation examples

Landmark analyses are already routinely used in ctDNA research (e.g.,^16^^,^^44^^,^^45^) due to their straightforward implementation in practice. For an example of a joint survival model implementation see Qiu et al.^46^ Garcia-Murillas et al. showcase how the hazard ratio changes when using a time-dependent Cox model.^26^ Unfortunately, we could not find any examples in the ctDNA literature in which absolute risks were estimated unbiasedly in the surveillance setting (i.e., never detected vs. ever detected). However, we provide an example of this using our simulated data with the extended Kaplan Meier approach (see Supplementary Fig. S1 and the provided SAS code in Supplementary material). Another valid approach to analyse the risk of ctDNA positive patients in this simulated data set would have been the clock-reset approach.^31^ However, we are not aware of any implementation of this approach in ctDNA studies, despite it being relatively straightforward: for patients who become positive their time until recurrence starts at the time of ctDNA detection. Instead of including the time until becoming detectable (yielding immortal time bias) the “survival clock is re-set” at this timepoint. Important note: for estimation of risk in ctDNA never detected patients, patients who became positive should not be excluded! They have to be censored at the time of becoming ctDNA detected. For more details, see also the supplementary section on immortal time bias (“Simple illustration of immortal time bias”).

A short summary of recommendations mentioned throughout this paper is listed in Table 2.

## Discussion

The rapid evolution of ctDNA research offers great promise but also poses persistent methodological challenges. The most critical gap is the limited awareness that ctDNA detection is inherently time-dependent, with major implications for proper statistical modelling. The relationship between longitudinal biomarkers and time-to-event outcomes is not straightforward, yet many studies apply conventional survival methods without adjustment. Even basic principles, such as accounting for censoring when estimating median lead times, are sometimes overlooked.

This commentary emphasises recurring analytical issues rather than offering an exhaustive catalogue of methods. Still, several priorities are clear: (i) predefine estimands aligned to clinical questions (ICH E9(R1)); (ii) estimate lead time with censoring/interval-censoring in mind; (iii) report time-dependent diagnostic metrics at prespecified horizons; and (iv) avoid immortal time bias via appropriate time-to-event strategies. Ultimately, only methodological sound analyses can clarify when and how acting on ctDNA meaningfully improves outcomes.

Lead time remains difficult to define (Fig. 1). Studies typically measure observed lead time between ctDNA detection and clinical recurrence, rather than true biological lead time. This observed interval varies with testing frequency and monitoring intensity. While retrospective studies report lead times of months to years, early breast cancer trials like c-TRAK TN^11^ and ZEST (NCT04915755; registration date: 2021-06-01) found many patients already had overt metastases at first ctDNA positivity, suggesting a minimal window for intervention.^11^ However, determining actual lead time requires statistical methods for handling interval censoring on both ends as it depends on ctDNA sampling frequency and disease monitoring density (Supplementary Fig. S3) but this topic was out of the scope of this paper. Only then can we optimise testing and trial designs (see also Supplement).

Another important methodological challenge relates to the substantial heterogeneity of imaging surveillance strategies across different tumour entities. For example, in early breast cancer, routine imaging for detection of distant metastases is generally not recommended in asymptomatic patients, whereas colorectal cancer guidelines commonly recommend regular CT-based surveillance during the first years after completion of therapy.^47^^,^^48^ These differences substantially influence the timing of clinical recurrence detection and, consequently, the apparent “lead time” associated with ctDNA-based MRD detection. Particularly outside prospective clinical trials with predefined imaging schedules, this introduces considerable potential for bias. Imaging examinations are often performed in an uncoordinated, indication-driven, and highly individualised manner. As a result, the interval between ctDNA positivity and radiological recurrence may be heavily influenced by differences in surveillance intensity rather than reflecting true biological differences in tumour progression. Additional research from the clinical community is necessary to explore the effects on ctDNA lead time. It is also noteworthy that these biases are not unique to ctDNA, but rather a general problem for prognostic longitudinal biomarkers in the surveillance setting.

A clear grasp of diagnostic performance is equally important. Measures such as sensitivity etc., are essential for evaluating clinical utility and differ across tumour types. However, these metrics are inherently time-dependent, varying with follow-up duration and relapse timing. Studies must move beyond static proportions and adopt analytical frameworks to yield interpretable clinical results. Appropriate statistical methods exist but remain underused in ctDNA research, making performance metrics hard to compare. Importantly, the field has already learnt that in time-to-event studies, reporting the complete Kaplan–Meier curve is more informative and appropriate than providing only single recurrence estimates. Similarly, journals and peer reviewers should request that the trajectories of these metrics across the entire follow-up period should be reported rather than reducing them to isolated summary values, which inevitably conceal important information. Through such an approach we may also gain new biological insights, similar to how understanding time to relapse transformed our understanding of breast cancer biology and risk stratification: for hormone receptor positive cancers, the risk of relapse is rather constant over time and therefore, we need very long follow up (maybe this is also true for sensitivity in hormone receptor positive breast cancer?). We also learnt that for aggressive diseases like triple negative breast cancer, we may only need 3–4 years of follow up to monitor most relapses. It seems plausible that for such aggressive diseases, the sensitivity and related metrics may also need only a shorter reporting period (perhaps 3 years).

Perhaps most challenging is the unbiased assessment of association between ctDNA and long-term outcomes. Standard survival methods, such as Kaplan–Meier and Cox models, are often misapplied due to immortal time bias issue. This can lead to misleading conclusions, with significant implications for clinical decision-making and patient care. Modern methods like landmark and joint survival models allow for more accurate assessment of ctDNA’s prognostic value. For example, a report by Garcia Murillas et al. from 2019 demonstrated that standard (biased) Cox models can underestimate the prognostic ability of ctDNA.^26^ They reported a HR of 25.2 from a time-dependent model, compared to a HR of “only” 16.7 from a “standard” Cox model.

Importantly, ctDNA studies sometimes have relapse focused endpoints which deliberately do not include deaths without prior recurrence as event of interest. Estimating rates and risks in these situations requires accounting for competing risks, and appropriate statistical methods must be used to obtain unbiased estimates (e.g., Aalen-Johansen estimator, Fine&Gray subdistribution hazards model^49^).

This paper provides an initial overview of ctDNA studies but does not address all complexities nor all pitfalls in depth. Important topics not covered include the prevalence dependence of PPV and NPV, or the potential proportional hazards violation when using the Cox model for ctDNA landmark analysis (i.e., positive patients usually have quite a high risk in the beginning that reduces to a similar risk as ctDNA negative patients), but also immortal time bias arising in other stratified analysis. Last but not least, we did not cover in this paper the different definitions for sensitivity and specificity that are covered elsewhere (e.g.,^20^). Many issues remain unresolved, and the suitability of various statistical methods depends on specific context. However, raising awareness of the key issues in the ctDNA community is an important first step toward improving the quality and interpretability of ctDNA studies (see Table 2). Future research should focus on defining and harmonising ctDNA lead time definitions, and the appropriate use of software for time-dependent estimation of performance metrics. Also, it will be important to determine which statistical methods most appropriately estimate the absolute risk associated with MRD detection in surveillance settings.

Future MRD studies should align lead-time analyses and molecular recurrence endpoints with established regulatory frameworks, including the ICH E9(R1) estimand framework, to ensure transparent handling of intercurrent events such as irregular ctDNA sampling, surveillance-related imaging differences, treatment initiation prior to clinical relapse, and competing risks. Such standardisation is essential for the interpretation, comparability, and potential regulatory qualification of ctDNA-based MRD endpoints. Importantly, the distinction between analytical validity, clinical validity, and clinical utility remains critical in this context. While many ctDNA assays already demonstrate high analytical sensitivity and specificity and substantial clinical validity for recurrence prediction, evidence for clinical utility — namely improved patient outcomes resulting from ctDNA-guided interventions — remains limited in many settings. Interpretation of analyses should therefore carefully distinguish between earlier molecular detection of recurrence and a true patient-relevant benefit associated with therapeutic intervention.”

Finally, although the paper and its examples focused on the ctDNA surveillance setting all of the reported pitfalls are inherently related to the analysis of prognostic longitudinal biomarker measurements and therefore are widely applicable in cancer research, especially in early cancer detection but also in metastatic settings.

In summary, ctDNA is a highly promising biomarker in oncology, but its full potential can only be realised through the rigorous application of appropriate statistical methods. Retrieving full insight into ctDNA’s usefulness relies on methodologically sound analyses that respect the time-dependent intricacies inherent to longitudinal biomarker data. We do hope that one day reporting of time-dependent sensitivity and specificity becomes the gold standard, just like Kaplan Meier plots are for time to recurrence endpoints.

## Outstanding questions

Key unresolved questions pertain to the influence of varying definitions of lead time, as well as the most suitable methods for estimating relapse risk in ctDNA surveillance settings, especially for patients with consistently positive vs. consistently negative ctDNA. Additionally, it will be crucial to determine whether insights can be gained from the time-dependency of sensitivity and specificity regarding the optimal sampling frequency and duration of follow up necessary to evaluate the performance of ctDNA assays. As more novel ultrasensitive methods emerge, all the relevant questions will have to be applied to specific technologies to allow future implementation.

## Contributors

Dominik Hlauschek: conceptualisation, data curation, formal analysis, investigation, methodology, project administration, resources, software, validation, visualisation, writing—original draft, writing– review & editing.

Marija Balic, Gabriel Rinnerthaler, Nadia Dandachi: conceptualisation, investigation, methodology, project administration, writing– review & editing, supervision.

Michael Gnant, Heather Parsons, Ellen Heitzer, Angela DeMichele: writing– review & editing, supervision.

All authors read and approved the final version of the manuscript.

## Data sharing statement

Simulated and published data sets were used. Simulated data are not available as stored data sets but can be re-generated with the SAS code that is provided in the Supplementary material. Additional SAS code can be shared upon request from the corresponding author (d.hlauschek@posteo.at).

The published I-SPY2 ctDNA data set can be accessed from the original publication by Magbanua et al. Cancer Cell. 2023; 41(6):1091–1102. e4. https://doi.org/10.1016/j.ccell.2023.04.008 Supplementary Table S1.

## Declaration of interests

Dominik Hlauschek: No conflicts of interests.

Gabriel Rinnerthaler: Grants or contracts from any entity: AstraZeneca, Daiichi Sankyo, Stemline; Consulting fees: Gilead; Support for attending meetings and/or travel: Daiichi Sankyo, Gilead, Roche, Servier; Participation on a Data Safety Monitoring Board or Advisory Board: AstraZeneca, BMS, Daiichi Sankyo, Eli Lilly, Gilead, Novartis, Pfizer, Stemline; Marija Balic: Grants or contracts from any entity: Astra Zeneca, Daiichi Sankyo, Novartis, Pfizer; Payment or honoraria for lectures, presentations, speakers bureaus, manuscript writing or educational events: Gilead, Stemline. Participation on a Data Safety Monitoring Board or Advisory Board: Gilead, Nadia Dandachi: No conflicts of interests.

Angela DeMichele: Grants or contracts from any entity: Pfizer, Novartis, Danaher, Genentech, Neogenomics; Payment or honoraria for lectures, presentations, speakers bureaus, manuscript writing or educational events: PER, Research to Practice; Participation on a Data Safety Monitoring Board or Advisory Board: Pfizer; Heather Parsons: Participation on a Data Safety Monitoring Board or Advisory Board: Exact Sciences, SAGA Diagnostics, Foresight Diagnostics, Daiichi-Sankyo, Gilead Sciences, Natera, Pfizer, Genentech, Illumina, Neogenomics; Michael Gnant: Consulting Fees: MSD, EliLilly, Novartis, Bayer, Menarini-Stemline, AstraZeneca; Payment or honoraria for lectures, presentations, speakers bureaus, manuscript writing or educational events: AstraZeneca, Daiichi Sankyo, EliLilly, EPQHealth, Novartis, PierreFabre; Payment for expert testimony: Veracyte; Support for attending meetings and/or travel: EliLilly, Novartis, Menarini-Stemline; Leadership or fiduciary role in other board, society, committee or advocacy group, paid or unpaid: ABCSG GmbH, ABCSG Res Serv GmbH; Ellen Heitzer: Grants or contracts from any entity: Illumina, Servier, PreanalytiX; Payment or honoraria for lectures, presentations, speakers bureaus, manuscript writing or educational events: Boehringer Ingelheim, Amgen, Incyte; Participation on a Data Safety Monitoring Board or Advisory Board: Roche, Astra Zeneca.
