# Supplementary material for: Common analysis pitfalls in longitudinal ctDNA studies: lead time, sensitivity and immortal time bias
Source: eBioMedicine. 2026 Jul 27;130:106407. doi: 10.1016/j.ebiom.2026.106407 (PMC13445507; doi:10.1016/j.ebiom.2026.106407)
Supplement: Supplementary file 1 [file mmc1.docx]

**Supplement:**

**Lead time:**

**Table S1. Biased median lead time according to increasing censoring rates.** 10.000 lead times were simulated from an exponential distribution (i.e., constant event rate) with a true median lead time of 12 months. In addition, censoring times were simulated independently also from an exponential distribution with varying rates. The second column of the table shows the naively estimated median lead time using just observed events.

| Censoring rate | Estimated median lead time using non-censored patients only |
| --- | --- |
| None | 12.0 months |
| Low (10% within 1 year) | 10.1 months |
| Middle (20% within 1 year) | 8.9 months |
| High (30% within 1 year) | 7.7 months |

**Lead time calculation example:**

We use a published data set from a ctDNA analysis in a lung cancer surveillance setting from Qiu et al^1^ to showcase how to calculate the median lead time using the Kaplan Meier approach in R and to contrast it with the naïve approach. The data set can be downloaded from <https://github.com/cancer-oncogenomics/ctDNA-dynamic-prediction-lung-cancer>. We used the Joint_model_data.csv data set. Specifically, we used the variables PatientID, Testdate (time of ctDNA test), Test_status (pos vs neg), and DFS (DFS time in months) as well as DFS_status (binary indicator of DFS event). Note: this data set is in long format meaning that each test result for a patient is in a new row, resulting in multiple observations per patient. See screenshot below for the first 2 patients for those selected variables:

e

This data set has 280 records and includes 89 patients, of whom 30 were ctDNA positive at any timepoint.

R example:

After importing the data set, we prepare the appropriate data set for Kaplan Meier estimation. For this, we filter the data set to retain only patients who were ctDNA positive at least once. Next, we derive the time of the first ctDNA positive test and the lead time as time from first positive test until the DFS event/censoring time (lead_time = DFS_time – first_positive_test).

Side note: As mentioned in the paper, this is just ONE approach to estimate the median lead time.

R-code:

\# loading the required packages

library(dplyr)

library(survival)

#importing data set from Qiu et al, NATURE communications 2021

Joint_model_data <- read.csv("path/Joint_model_data.csv") #adapt the path accordingly.

\# Kaplan Meier data set preparation

km_data <- Joint_model_data |>

group_by(PatientID) |>

\# Filter for patients who have at least one positive test

filter(any(Test_status == "Pos")) |>

summarise(

first_positive_test = min(TestDate[Test_status == "Pos"], na.rm = TRUE),

DFS_time = first(DFS),

DFS_status = first(DFS_status),

.groups = "drop"

) |>

\# Calculate lead time and filter for valid observations

mutate(lead_time = DFS_time - first_positive_test) |>

filter(lead_time >= 0)

As already mentioned, this data set includes 30 patients who were ctDNA positive at some point during follow up. Of these, 22 (73.3%) experienced a recurrence during follow-up. To calculate the median lead time for this population, the 8 patients who were censored with last follow up need to be considered appropriately, because their lead time is at least the time from positive test until the last follow up.

Note: DFS_status represents the event status at the end of follow up (event=1, censored=0).

R-code:

\# Fit the Kaplan-Meier model

km_fit <- survfit(Surv(lead_time, DFS_status) ~ 1, data = km_data)

summary(km_fit)$table #this gives the table of estimates including median time and 95% confidence intervals

From this analysis we can see that the median lead time until DFS event = 8.9 months (95% CI = 7.5 to 11.6).

In contrast: a naïve median lead time analysis ignoring the censored patients (n=22) would have given a (biased) median lead time estimate of 7.6 months (see code below).

R-code:

#naive median lead_time in DFS_status = 1 patients only

median(km_data$lead_time[km_data$DFS_status == 1])

Last but not least, we can also visualize the lead time from the first ctDNA positive test until the DFS event using a standard Kaplan Meier plot.

\# use survminer for a professional ggplot-based survival plot

library(survminer)

KMplot <- ggsurvplot(km_fit, data = km_data, fun = "event", risk.table = TRUE, conf.int = TRUE, xlab = "Lead Time (Time from first positive test to event)", ylab = "DFS", ggtheme = theme_minimal())

KMplot$plot <- KMplot$plot +

\# Horizontal segment from the y-axis to the median

geom_segment(aes(x = 0, y = 0.5, xend = median_km_lead_time, yend = 0.5),

linetype = "dashed", color = "black") +

\# Vertical segment from the x-axis to the median

geom_segment(aes(x = median_km_lead_time, y = 0, xend = median_km_lead_time, yend = 0.5),

linetype = "dashed", color = "black")

The Kaplan Meier plot below shows that some patients have lead times greater than 12 months, but it can also be seen from the small tick marks that some patients have not yet experienced the event. In particular, there are 5 patients with a lead time of at least 12 months.

**Important note:** This showcase was intended to illustrate how median lead time can be calculated using the Kaplan Meier method. In practice, there are additional considerations that depend on the study and data set at hand. For example, one may not want to use the first positive ctDNA test if it is followed by a ctDNA negative test (see also discussion in the main paper). In this case, only patients who were ctDNA positive before the DFS event/censoring would be included in the Kaplan Meier analysis. In this small data example 19 patients would remain, and the median lead time would shrink to about 7 months. Furthermore, one may want to censor patients who became ctDNA negative after a positive test to estimate the hypothetical median lead time had they not become negative. Also, if there are several missing ctDNA tests during follow up one may want to consider those (using imputation or censoring at the time of a missed test).

**Diagnostic Performance Metrics:**

**Definitions:**

### **Table S2. Definitions of key diagnostic performance metrics for ctDNA assays**

| **Metric** | **Definition (conceptual)** | **Interpretation / Notes** |
| --- | --- | --- |
| **Sensitivity** | Fraction of ctDNA-positive patients among those who relapsed within a defined time interval | Reflects the assay’s ability to correctly identify patients who will experience recurrence; may decrease with longer follow-up as late relapses occur after ctDNA negativity. |
| **Specificity** | Fraction of ctDNA-negative patients among those who remained recurrence-free within a defined time interval | Indicates the assay’s ability to correctly classify patients who will not relapse; may increase over time as more true negatives are confirmed. |
| **Positive Predictive Value (PPV)** | Fraction of patients who relapsed within a defined time interval among all ctDNA-positive individuals | Represents the probability that ctDNA positive patients relapse; increases with follow-up as more ctDNA-positive patients relapse. |
| **Negative Predictive Value (NPV)** | Fraction of patients who remained recurrence-free within a defined time interval among all ctDNA-negative individuals | Represents the probability that ctDNA negative patients remain relapse-free; decreases over time as additional late recurrences occur. |

**Formulas:**

All 4 metrics can be formulated as conditional probabilities depending on time *t*. Furthermore, Sensitivity and Specificity can be rewritten using Bayes theorem and the law of total probability (see also ^2^):

$$Sensitivity \left( t \right)= P\left( ctDNA= positive \right| Relapse before t)=\frac{P\left( ctDNA= positive \right)* P\left( Relapse before t \right| ctDNA=positive)}{P\left( relapse before t \right)} = \frac{P\left( ctDNA= positive \right)*PPV\left( t \right)}{P\left( ctDNA=positive \right)*PPV\left( t \right)+P\left( ctDNA=not detected \right)*\left( 1-NPV\left( t \right) \right)}$$

$$Specificity \left( t \right)=P\left( ctDNA=not detected \right| No relapse before t)=\frac{P\left( ctDNA=not detected \right)*P\left( No relapse before t \right| ctDNA=not detected)}{P(No relapse before t)}=\frac{P\left( ctDNA=not detected \right)*NPV\left( t \right))}{P\left( ctDNA=positive \right)*(1-PPV\left( t \right))+P\left( ctDNA=not detected \right)*NPV(t)}$$

$$Positive Predictive Value \left( t \right)=P\left( Relapse before t \right| ctDNA= positive)$$

$$Negative Predictive Value \left( t \right)=P\left( No relapse before t \right| ctDNA=not detected)$$

**Estimation:**

PPV and NPV can simply be estimated using Kaplan Meier methodology:

$$\hat{PPV\left( t \right)}={1-\hat{KM}}_{positive}(t)$$

$$\hat{NPV\left( t \right)}=\hat{KM}_{not detected}(t)$$

Whereas $\hat{KM}_{positive}(t)$ refers to the Kaplan Meier estimator at time *t* in ctDNA positive patients and $\hat{KM}_{not detected}(t)$ refers to the Kaplan Meier estimator at time t in ctDNA non-detected patients.

Sensitivity and Specificity can simply be estimated by plugging in the estimators for PPV and NPV as well as the fraction of ctDNA positive (i.e., $\hat{\pi}_{positive}$) and ctDNA non-detected patients (i.e., $\hat{\pi}_{negative}=1- \hat{\pi}_{positive}$):

$$\hat{Sensitivity(t)}= \frac{\hat{\pi}_{positive}*\hat{PPV\left( t \right)}}{\hat{\pi}_{positive}*\hat{PPV\left( t \right)}+\hat{\pi}_{negative}*\left( 1-\hat{NPV\left( t \right)} \right)}$$

$$\hat{Specificity(t)}= \frac{\hat{\pi}_{negative}*\hat{NPV\left( t \right)}}{\hat{\pi}_{positive}*(1-\hat{PPV\left( t \right))}+\hat{\pi}_{negative}*\hat{NPV\left( t \right)}}$$

SAS Code for calculating these metrics:

ods graphics on;

*importing data set (the original file was just prepared in a way to make the import flawless [i.e. column names were sligthly adapted]);

proc import datafile="path\I-Spy 2 ctDNA data_prep.xlsx"

dbms=xlsx replace out=ispy;

run; *adapt path accordingly;

data ispy; set ispy;

DRFS_time = DRFS_time / 365.25; *converting days to years;

run;

*naive sensitivity and specificity (not considering censoring and time dependency);

proc sort data=ispy; by descending drfs descending ctDNA_TP3; run;

proc freq data=ispy order=data;

table ctDNA_TP3*DRFS / senspec;

run;

*** Option A to calculate time-dependent sensitivity and specificity using ROCOPTIONS in PROC PHREG;

proc phreg data=ispy plots=roc rocoptions(method=km at=(1 2 3 4 5) outroc=roc);

class ctDNA_TP3(Ref="Negative");

model DRFS_time*DRFS(0) = ctDNA_TP3 / rl;

run;

*** Option B to calculate time-dependent sensitivity and specificity using the plug in formulas based on Kaplan Meier estimation and Bayes Rule

see supplemental material for the formulas;

*macro to derive the metrics;

%macro Metrics(indata, ctDNAvar, timevar, censvar); *macro call inputs are the data set, the ctDNA variable, the time to event and censoring variables;

ods exclude all;

*calculation of Kaplan Meier rates for 0 to 5 years in ctDNA positive and negative patients;

ods output censoredsummary=cs;

proc lifetest data=&indata. plots=survival(atrisk) timelist=(0 to 5 by 1) outs=KMrates reduceout;

time &timevar.*&censvar.(0);

strata &ctDNAvar. / notest;

run;

*extract number of ctDNA positive and negative patients;

proc sql noprint; select total into :npos from cs where &ctDNAvar. eq "Positive"; quit;

proc sql noprint; select total into :nneg from cs where &ctDNAvar. eq "Negative"; quit;

*extract number of total patients;

proc sql noprint; select total into :ntotal separated by "" from cs where missing(&ctDNAvar.); quit;

*sort and transpose data set with Kaplan Meier rates to have columns instead of rows with the timepoints;

proc sort data=kmrates; by timelist; run;

proc transpose data=KMrates out=Metrics prefix=KM_; var survival; id &ctDNAvar.; by timelist; run;

*calculation of sensitivity and specificity;

data Metrics; set Metrics;

format ctDNAprev PPV NPV ProbEvent Sensitivity Specificity percent8.1;

ctDNAprev = &npos. / (&npos.+&nneg.); *ctDNA positive prevalence;

PPV = 1-KM_Positive;	*Positive and negative predictive value are just the Kaplan Meier estimates;

NPV = KM_Negative;

ProbEvent = PPV * ctDNAprev + (1-NPV) * (1-ctDNAprev); *Probability of an event in the entire cohort (=denominator in formula below);

*sensitivity and specificity;

if timelist ne 0 then Sensitivity = ctDNAprev * PPV / ProbEvent; *for formula explanation see supplementary material;

else Sensitivity = ctDNAprev; *exception rule: at time 0 sensitivity = 0, because PPV is 0. However, imputing ctDNA prevalance for this case just avoids an akward plot, but this can also be removed;

Specificity = (1-ctDNAprev) * NPV / (1-ProbEvent); *for formula explanation see supplementary material;

run;

*transpose metrics data set to have metrics in rows for plot;

proc transpose data=metrics out=metricsT; var PPV NPV Sensitivity Specificity; by timelist; run;

ods exclude none;

*Plotting all diagnostic metrics in one plot over time with PROC SGPLOT;

proc sgplot data=metricsT;

where timelist gt 0;

scatter x=timelist y=survival / group=_name_ datalabel;

series x=timelist y=survival / group=_name_ name="leg";

keylegend "leg" / title="Metric";

xaxis offsetmin=0.05 offsetmax=0.05 grid label="Years from patient consent";

yaxis values=(0 to 1 by 0.1) grid label="Diagnostic performance metrics [%]";

inset "n=&ntotal." / border;

run;

%mend metrics;

***Diagnostic metrics for different ctDNA timepoints;

*baeseline timepoint;

%Metrics(ispy, ctdna_tp0, DRFS_time, DRFS)

*baseline timepoint by breast cancer subtype;

%Metrics(ispy(where=(subtype eq "HR+/HER2-")), ctdna_tp0, DRFS_time, DRFS)

%Metrics(ispy(where=(subtype ne "HR+/HER2-")), ctdna_tp0, DRFS_time, DRFS)

*other timepoints;

%Metrics(ispy, ctdna_tp1, DRFS_time, DRFS)

%Metrics(ispy, ctdna_tp2, DRFS_time, DRFS)

*pre-surgical timepoint -> same figure as in main paper;

%Metrics(ispy, ctdna_tp3, DRFS_time, DRFS)

**Artificial censoring simulation using I-SPY2 data:**

To illustrate the influence of different censoring patterns on the naively estimated Sensitivity etc., we used a small simulation study using the I-SPY2 data and introducing additional censoring times. Time to artificial censoring was simulated for every patient using a constant censoring rate (i.e., exponential distributed times). The following 3 scenarios were investigated:

- Scenario 1: yearly censoring rate = 5% for all patients
- Scenario 2: yearly censoring rate = 10% for all patients
- Scenario 3: yearly censoring rate = 20% for ctDNA positive patients and 5% for ctDNA non-detected patients.

The following steps were performed for each scenario:

1) Observed time-to-events (DRFS) of the I-SPY2 data were censored in case the artificial censoring time was smaller (i.e., the minimum of both times was taken) and the event indicator was updated accordingly.
2) Diagnostic metrics were calculated using the new time-to-events using simple proportions for Table 2. Furthermore, the four metrics were also derived using (unbiased) Kaplan Meier methodology. See formulas on page 1 of supplementary file.
3) The process was repeated 5000 times to reduce the random variability to a minimum (i.e., averages of all simulations up to the first decimal point remained very consistent).
4) Averages were calculated over all 5000 simulations.

**Outcomes of the Illustration example using I-SPY2 data**

As shown in Table 2 in the main manuscript, all four (naively estimated) metrics depend on the censoring distribution. On the other hand, Table S3 below shows that the unbiased time-dependent estimates are not affected by the censoring distribution.

**Table S3:** Diagnostic performance metrics of pre-surgical ctDNA status using the I-SPY2 data for three different time-points. The same additional artificial censoring patterns as introduced for Table 2 in the main paper were used.

| **Time-point (from patient consent)** | **Metric** | **Estimate**  **(no additional censoring)** | **Artificial yearly censoring rate  for ctDNA positive vs non-detected patients** | | |
| --- | --- | --- | --- | --- | --- |
|  |  |  | **5% / 5%** | **10% / 10%** | **20% / 5%** |
| 1 year | Sensitivity | 72,2% | 72,2% | 72,2% | 72,1% |
|  | Specificity | 88,1% | 88,1% | 88,1% | 88,1% |
|  | PPV | 34,2% | 34,2% | 34,2% | 34,2% |
|  | NPV | 97,4% | 97,4% | 97,4% | 97,4% |
| 2 years | Sensitivity | 53,5% | 53,6% | 53,6% | 53,5% |
|  | Specificity | 90,6% | 90,6% | 90,6% | 90,6% |
|  | PPV | 52,6% | 52,6% | 52,6% | 52,6% |
|  | NPV | 90,9% | 90,9% | 90,9% | 90,9% |
| 3 years | Sensitivity | 43,0% | 43,0% | 43,0% | 42,9% |
|  | Specificity | 92,5% | 92,6% | 92,6% | 92,6% |
|  | PPV | 66,9% | 66,9% | 67,0% | 66,9% |
|  | NPV | 82,2% | 82,2% | 82,2% | 82,2% |

**Simulations for immortal time bias illustration**

A simulation was run with 10000 patients assuming constant relapse rate (i.e., exponential distribution) to illustrate immortal time bias in relapse-free rate estimation for persistently ctDNA non-detected patients and for estimation for patients becoming ctDNA positive.

The following steps were taken:

1) At baseline it was assumed that 10% are already ctDNA positive and for the rest time to ctDNA detection was simulated with a detection rate of 40% within 5 years (constant rate over time).
2) Time until relapse was simulated using the approach/formula for time-dependent exponentially distributed survival from Austin^3^ (formula (1)).
   a. Relapse-free rate of 90% within 5-years for patients where ctDNA was not detected prior to the relapse.
   b. Relapse-free rate increased 10-fold (hazard ratio = 20) after ctDNA detection.
3) A patient was considered ctDNA positive if time until detection was smaller than the simulated time until relapse.
4) No censoring was performed except that follow up was censored for all event-free patients at 5-years.
5) Naïve Kaplan Meier estimation was performed to demonstrate the biased estimation of the relapse risk in patients without a ctDNA positive test (i.e., persistently ctDNA non-detected) and in patients being/becoming ctDNA positive before relapse. Time until relapse was not modified.
6) Extended Kaplan Meier estimation was performed to demonstrate the unbiased estimation of relapse risk in ctDNA persistently negative and persistently positive patients. For that, the data set was modified (counting process style) to enable use of PROC PHREG in SAS. For patients becoming ctDNA positive before relapse two data rows had to be created: one for the time period before ctDNA detection and one after that.

For details see the SAS code below and Figure S1.

The full SAS code to replicate the simulation (including the seed that was used for the figure) is shown below:

SAS Code for immortal time bias simulations:

ods graphics on;

%let out=path; *specify path for output file;

*simulation parameters;

%let n=10000; *sample size;

%let lambda = (-log(0.90)/5); *lambda parameter for relapse-free times (90% event free at 5 years);

%let ctDNAHR = 20; *time-dependent HR for ctDNA positive patients;

data sim;

call streaminit(123); *seed;

do i=1 to &n.;

*simulating ctDNA positivity: 10% are positive at beginning of follow up and the rest gets positive with 40% probability during follow up;

if rand("bernoulli", 0.1) then ctDNAposTime = 0;

else ctDNAposTime = rand("exponential", 1/(-log(0.6)/5)); *time of first ctDNA positive test (40% within first 5 years);

*time-dependent random relapse-free times acc. to Austin 2012 (exponential distribtion, formula (1));

uniformTime = rand("uniform"); *uniform random number;

if -log(uniformTime) lt (&lambda. * ctDNAposTime) then RelapseTime = -log(uniformTime) / (&lambda.);

else RelapseTime = (-log(uniformTime) - &lambda.*ctDNAposTime + &lambda.*&ctDNAHR.*ctDNAposTime) / (&lambda.*&ctDNAHR.);

*observed times and event indicator;

ObsTime = min(RelapseTime,5); *censor everyone at 5 years;

CensInd = ObsTime = RelapseTime; *0 for no event and 1 for event;

*indicator for ctDNA detected during observed time;

ctDNA = "Not detected"; if ctDNAposTime lt ObsTime then ctDNA = "Positive";

output;

end;

run;

*naive Kaplan Meier analysis by observed ctDNA status;

ods output SurvivalPLot = KMplot0;

proc lifetest data=sim plots=survival(atrisk cl) timelist=5;

time ObsTime*CensInd(0);

strata ctDNA;

run;

*prepare data for plot;

data KMplot; set KMplot0;

format Survival sdf_lcl sdf_ucl TrueSurvNeg TrueSurvPos percent8.1;

if stratum eq "Not detected" then do;

TrueSurvNeg = exp(-Time*&lambda.); *true survival rate due to simulation parameters in ctDNA negative patients;

group = "Naive Kaplan Meier for ctDNA negative";

end;

else do;

TrueSurvPos = exp(-Time*&lambda.*&ctDNAHR.); *true survival rate due to simulation parameters in ctDNA positive patients;

group = "Naive Kaplan Meier for ctDNA positive";

end;

label TrueSurvNeg = "True Relapse-free Survival ctDNA negative" TrueSurvPos = "True Relapse-free Survival ctDNA positive" ;

run;

*plot;

ods graphics / outputfmt=pdf imagename="Figure 3 Immortal time bias in naive Kaplan Meier estimation" noborder;

ods listing gpath="&out." image_dpi=300;

proc sgplot data=KMplot;

band x=time lower=sdf_lcl upper=sdf_ucl / transparency=0.7 type=step group=group;

step x=time y=Survival / name="leg1" group=group lineattrs=(pattern=1);

series x=time y=TrueSurvpos / lineattrs=(pattern=2 color=black) name="leg2";

series x=time y=TrueSurvneg / lineattrs=(pattern=4 color=black) name="leg3";

xaxistable atrisk / x=tatrisk class=group colorgroup=group;

yaxis label="Relapse-free probability" values=(0 to 1 by 0.1);

xaxis label="Years";

keylegend "leg1" "leg2" "leg3" / location=inside position=bottomleft;

run;

ods pdf close;

***extended Kaplan Meier analysis ***;

*adapting the data set into long format (counting process);

data simadapted; set sim;

if ctDNA eq "Positive" then do; *for positive patients two rows are necessary: one for the time until ctDNA detection and one after that;

starttime = 0; endtime = ctDNAposTime; cens = 0; strata = "Not detected"; output; *time period from start of follow up until ctDNA positivity;

starttime = ctDNAposTime; endtime = obstime; cens = CensInd; strata = "Positive"; output; *time period from ctDNA positivity until event/censoring;

end;

else do; *for ctDNA negative patients only one row is needed;

starttime = 0; endtime = obstime; cens = censInd; strata = "Not detected"; output;

end;

run;

*Extended KM method using PROC PHREG and BASELINE (the same procedure could be used with strata as covariate - assuming proportional hazards);

proc phreg data=simadapted;

model (starttime,endtime)*cens(0) = ; *counting process notation;

strata strata;

baseline out=SurvEstimates survival=_all_ / method=PL; *method = Product limit to get survival estimates for the two strata;

run;

*prepare data for plot;

data SurvEstimates; set SurvEstimates;

format Survival lowersurvival UpperSurvival TrueSurvNeg TrueSurvPos percent8.1;

if strata eq "Not detected" then do;

TrueSurvNeg = exp(-endtime*&lambda.); *true survival rate due to simulation parameters in ctDNA negative patients;

group = "Extended Kaplan Meier for ctDNA negative";

end;

else do;

TrueSurvPos = exp(-endtime*&lambda.*&ctDNAHR.); *true survival rate due to simulation parameters in ctDNA positive patients;

group = "Extended Kaplan Meier for ctDNA positive";

end;

label TrueSurvNeg = "True Relapse-free Survival ctDNA negative" TrueSurvPos = "True Relapse-free Survival ctDNA positive" ;

run;

ods graphics / outputfmt=pdf imagename="Figure Supplement Immortal time bias_extended KM" noborder;

ods listing gpath="&out." image_dpi=300;

proc sgplot data=SurvEstimates;

band x=endtime lower=lowersurvival upper=uppersurvival / transparency=0.7 type=step group=group;

step x=endtime y=Survival / name="leg1" group=group lineattrs=(pattern=1);

series x=endtime y=TrueSurvpos / lineattrs=(pattern=2 color=black) name="leg2";

series x=endtime y=TrueSurvneg / lineattrs=(pattern=4 color=black) name="leg3";

yaxis label="Relapse-free probability" values=(0 to 1 by 0.1);

xaxis label="Years";

keylegend "leg1" "leg2" "leg3" / location=inside position=bottomleft;

run;

ods pdf close;

**Figure S1** Extended Kaplan Meier approach for simulated data to prevent immortal time bias

**Simple illustration of immortal time bias**

Figure S2 illustrates immortal time bias using two patients as an example. Both patients start ctDNA negative after definitive surgery (Figure S2a). Patient 1 remains negative until recurrence and patient 2 becomes ctDNA positive before recurrence. This creates four distinct time periods to consider during analysis (labeled 1-4 in Figure S2a). Panels b and c show common naïve strategies for estimating relapse risk in ctDNA non-detected (b) and detected patients (c).

b) Naïve analysis of ctDNA negative patients: A common approach is to exclude patients who become ctDNA detected at any time (shown with the purple crosses). However, this removes patient 2 and omits time period 3 from analysis, when the patient was ctDNA negative and recurrence-free. Excluding such intervals can lead to an *overestimated* risk for ctDNA non-detection. Proper statistical methods should include these negative, event-free intervals (time-period 3).

c) Naïve analysis of ctDNA positive patients: In this approach, all patients with no detectable ctDNA are excluded (time periods 1 and 2 are removed) and only patients who become positive prior recurrence are included. A common mistake is to classify these patients as ctDNA positive from the start of follow-up (here definitive surgery), rather than from the time of actual detection. This misclassifies time period 3 as ctDNA positive time as shown in red in Figure S2 c). Because recurrence cannot occur before ctDNA is first detected, this introduces immortal/guarantee time bias. As a result, the recurrence-free interval of ctDNA positive patients is artificially prolonged and the association between ctDNA positivity and recurrence is *underestimated*. Appropriate statistical methods account for this by correctly assigning only the post-ctDNA detection period to positive patients (period 4).

**Figure S2** Simple illustration of immortal time bias in ctDNA analysis

**Methods to avoid immortal time bias**

The following table S4 shows statistical methods that – when used appropriately – avoid immortal time bias (ITB) when estimating associations between ctDNA detected and non-detected patients. This table is not meant to be an exhaustive list of all available methods.

**Table S4:** Statistical methods for estimating associations of ctDNA with outcome

| **Method** | **Application area** | **(Some) implementation details** | **(Some) things to avoid** | **Example result** |
| --- | --- | --- | --- | --- |
| Kaplan Meier | Absolute risk estimation | -) An appropriate time origin has to be used (e.g. first ctDNA test result).  -) Kaplan Meier curves for ctDNA groups are created. | Using ctDNA test results from after baseline (=time 0) 🡪 ITB | Patients with positive ctDNA test results at diagnosis have a 3-year Recurrence rate of 50%. |
| Landmark^4,5^ | Absolute (conditional) Risk estimation | -) Appropriate landmark timepoint(s) have to be chosen.  -) Groups of patients based on ctDNA information from before landmarks are created  -) Kaplan Meier curves for patients who were event free at the landmark are created. | Using ctDNA test results from after the landmark 🡪 ITB | Patients who were ctDNA positive at baseline and cleared their ctDNA within 6 months (landmark) and where event free at 6 months have a 5-year Recurrence rate of 10% in the future (i.e., 5-years from landmark). |
| Clock-reset^6^ | Absolute risk estimation in patients who switch ctDNA status | -) At the time of ctDNA switch the “survival clock” is reset.  -) Kaplan Meier curves are created with the new time-scale.  -) check semi-Markov assumption if a causal group comparison is warranted. | -) Using ctDNA test results that became available after clock-reset timepoint 🡪 ITB  -) when comparing groups with different timescale be careful if semi-Markov assumption is violated^1^. | Patients have a 5-year risk of recurrence of 50% after becoming ctDNA positive. |
| Simon- Makuch^7^ / Extended Kaplan Meier^8,9^ | Absolute risk estimation in patients who switch ctDNA status | -) Patients changing their ctDNA status are censored at that time and “switch” the group.  -) At risk sets must be updated accordingly when patients switch ctDNA. | -) interpreting the results as simple Kaplan Meier estimates  -) Not updating the risk sets appropriately 🡪 ITB | The recurrence rate of patients who never became ctDNA positive was 5% at 10 years. |
| Time-dependent Cox model^5,10^ | Relative rate estimation | -) ctDNA status is being treated as time-dependent  -) time periods need to be assigned to ctDNA negative vs positive  -) Cox model with time-dependent ctDNA status | -) treating a time-dependent covariate as “fixed” baseline covariate 🡪 ITB  -) ignoring the proportional hazards assumption | The recurrence rate of patients who are ctDNA positive during follow up is 5.3 times the recurrence rate of patients who are ctDNA negative at the same time (time-dependent Hazard ratio = 5.3). |
| Joint survival model^11-14^ | Association of longitudinal ctDNA status/ trajectories with outcome | -) ctDNA status changes or ctDNA level changes over time are modeled with the repeated measures component of the joint model  -) time to event part is incorporated into the analysis with the survival model component | -) inclusion of longitudinal ctDNA results recorded after the event  -) starting with the most complex model 🡪 avoid overfitting | Per 1% increase in ctDNA tumor fraction the recurrence rate increases by 35%. |

**1) The risk of the groups may appear different because they are at different times in their disease history (e.g., 1-year from diagnosis vs 3-years from diagnosis), and not because of a different ctDNA status.**

**Observed versus true lead time of ctDNA**

The true lead time of ctDNA refers to the time ctDNA is traceable in the blood and the actual occurrence of metastasis. However, in practice we can only measure the observed time from a ctDNA positive test until the detection of metastasis, which is not the same, as shown below. Figure S3 shows a timeline for a patient with no ctDNA in the blood in the beginning which is also confirmed by a negative ctDNA test. At some point, ctDNA becomes traceable in the blood indicated by the switch from green to red. However, ctDNA is only detected during the next scheduled ctDNA test. Also shown in Figure S3 are two timepoints of Computer tomography (CT)/ Magnet resonance imaging (MRI) scans. Similarly, the patient has an initial negative CT/MRI scan, but metastases develop later and are detected only at the next scan. We can distinguish between observed lead time (the time from the positive test until the scan showing signs of metastasis) and the true lead time of ctDNA. The actual lead time of ctDNA can be estimated only when it is recognized that the data are subject to interval censoring. All we know is that the true timepoint of traceable ctDNA is somewhere between the negative and positive ctDNA test (1^st^ interval) and the true time of recurrence is somewhere between the two CT/MRI scans (2^nd^ interval).

**Figure S3** observed vs true lead time of ctDNA.

**References**

1.	Qiu B, Guo W, Zhang F, et al: Dynamic recurrence risk and adjuvant chemotherapy benefit prediction by ctDNA in resected NSCLC. Nature Communications 12:6770, 2021

2.	Antolini L, Valsecchi MG: Performance of binary markers for censored failure time outcome: nonparametric approach based on proportions. Statistics in Medicine 31:1113-1128, 2012

3.	Austin PC: Generating survival times to simulate Cox proportional hazards models with time-varying covariates. Stat Med 31:3946-58, 2012

4.	Gleiss A, Oberbauer R, Heinze G: An unjustified benefit: immortal time bias in the analysis of time-dependent events. Transplant International 31:125-130, 2018

5.	Putter H, van Houwelingen HC: Understanding Landmarking and Its Relation with Time-Dependent Cox Regression. Stat Biosci 9:489-503, 2017

6.	Bernasconi DP, Rebora P, Iacobelli S, et al: Survival probabilities with time-dependent treatment indicator: quantities and non-parametric estimators. Stat Med 35:1032-48, 2016

7.	Simon R, Makuch RW: A non-parametric graphical representation of the relationship between survival and the occurrence of an event: Application to responder versus non-responder bias. Statistics in Medicine 3:35-44, 1984

8.	Smith AR, Goodrich NP, Beil CA, et al: Graphical representation of survival curves in the presence of time-dependent categorical covariates with application to liver transplantation. Journal of Applied Statistics 46:1702-1713, 2019

9.	Jay M, Betensky RA: Displaying survival of patient groups defined by covariate paths: Extensions of the Kaplan-Meier estimator. Statistics in Medicine 40:2024-2036, 2021

10.	Therneau T, Crowson C, Atkinson E: Using time dependent covariates and time dependent coefficients in the cox model. Survival vignettes 2:1-25, 2017

11.	Ibrahim JG, Chu H, Chen LM: Basic concepts and methods for joint models of longitudinal and survival data. J Clin Oncol 28:2796-801, 2010

12.	Philipson P: joineR: Joint Modelling of Repeated Measurements and Time-to-Event Data,

13.	Rizopoulos D: JM: Joint Modeling of Longitudinal and Survival Data,

14.	Rizopoulos D: The R Package JMbayes for Fitting Joint Models for Longitudinal and Time-to-Event Data Using MCMC. Journal of Statistical Software 72:1 - 46, 2016
